# White Globe Appearance–Like Findings Indicating Intralymphatic Cancer Involvement Beneath the Epithelium in Gastric Cancer

**DOI:** 10.1155/2024/8504987

**Published:** 2024-10-19

**Authors:** Hiroki Maruyama, Taku Yamagata, Yoshihide Kanno, Takeshi Shimizu, Takuho Itasaka, Fumiyoshi Fujishima, Takashi Sawai, Kei Ito

**Affiliations:** ^1^Department of Gastroenterology, Sendai City Medical Center, Miyagi, Japan; ^2^Department of Pathology, Tohoku University Hospital, Miyagi, Japan; ^3^Department of Pathology, Sendai City Medical Center, Miyagi, Japan

**Keywords:** early gastric cancer, lateral spread, lymphatic invasion, magnifying endoscopy, narrow-band imaging

## Abstract

A 75-year-old female was diagnosed with a type 0-I, moderately differentiated, early gastric carcinoma on the posterior wall of the middle gastric body during esophagogastroduodenoscopy (EGD). Several small whitish structures, referred to as white globe appearances (WGAs), were noted on the oral side outside the demarcation line of the cancerous protrusion. Although this area was flat without cancerous mucosal changes on the surface, subepithelial cancer extension was suspected. The histopathology of the resected specimen revealed that the carcinoma with submucosal invasion had significant lymphatic invasion with submucosal lateral extent along lymphatic vessels. In some areas, the carcinoma within the lymphatic vessels regressed from the submucosal layer towards the mucosal lamina propria, penetrating the muscularis mucosas. The intralymphatic carcinoma reaching just beneath the epithelium was considered to manifest WGA features during endoscopy.

## 1. Introduction

The usefulness of endoscopic findings of white globe appearances (WGAs) in magnifying endoscopy for diagnosing early gastric cancer has been reported [1, 2]. WGAs are often seen in the marginal area of gastric cancers. However, in the present case, WGA-like findings were found outside the superficial demarcation line of an early-stage gastric cancer. Pathological evaluation of a surgically resected specimen revealed that the tumor nests in lymphatic vessels laterally extended within the submucosal layer with retrograde penetration toward the mucosal lamina propria. The intralymphatic carcinoma reaching just beneath the epithelium was recognized as WGA-like findings during endoscopy, manifesting small white spherical structures.

## 2. Case Report

**Figure 1:**
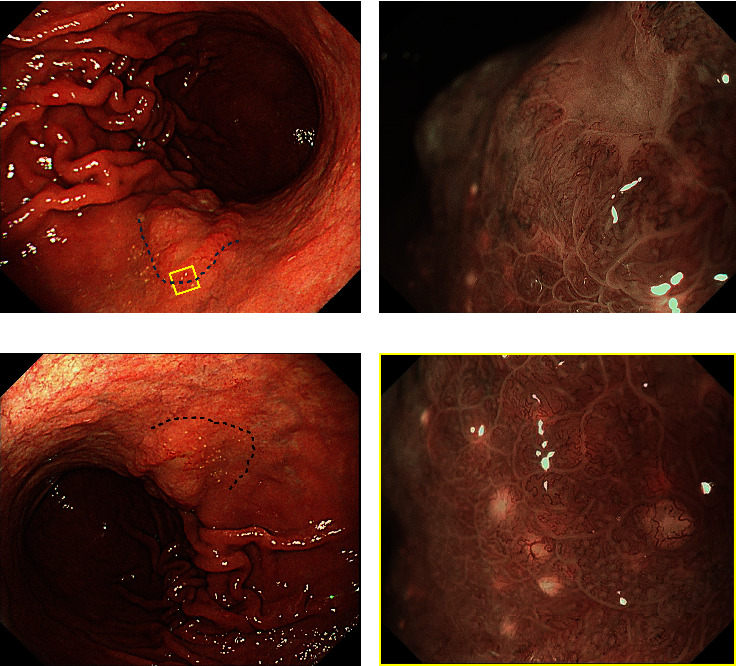
EGD images: (a) white light imaging revealing a 25-mm, elevated, slightly reddish lesion on the posterior wall of the gastric middle body. The edge of the elevating lesion is highlighted with a blue dotted line; (b) magnifying NBI for the marginal area of the depression at the center of the protrusion. Pronounced glandular duct structures and bent, tortuous vessels were observed; (c) scattered white dots were observed within a 10-mm wide area towards the oral side from the protrusion. The black dotted line indicates the area where the white dots are scattered; (d) magnifying NBI within the yellow box shown in 1(a). Small white spherical structures with microvessels crossing over them were observed.

A 75-year-old woman, without significant complications or past medical history, was referred to our hospital due to complaints of acid reflux. No proton pump inhibitor or potassium-competitive acid blocker was taken. Esophagogastroduodenoscopy (EGD) revealed a 25-mm, faint erythematous lesion on the posterior wall of the middle gastric body with background atrophy classified as Kimura–Takemoto Classification O-III. The lesion was slightly firm and grossly elevated and had a depressed area at its center (Figure 1(a)). The edge of the elevating lesion is highlighted with a blue dotted line. Magnifying endoscopy with narrow-band imaging (M-NBI) for the margins of the depression showed indistinct glandular duct structures and strongly tortuous vessels, indicating a moderately to poorly differentiated adenocarcinoma (Figure 1(b)). The biopsy of the primary protruding lesion revealed irregular clusters of glandular ducts composed of atypical cells with conspicuous nuclear atypia, and ductal formations were unclear in some lesions. A diagnosis of moderately to poorly differentiated adenocarcinoma was made.

Within a 10-mm wide area towards the oral side from the superficial demarcation line of the carcinoma, wherein no vascular and structural atypia were observed, scattered tiny white dots < 1 mm were noted (Figure 1(c)). Under magnifying endoscopy, each white dot appeared round and slightly spherical, lacking glandular structures, with microvessels crossing over them, fully demonstrating the characteristics of the previously reported WGA [1] (Figure 1(d)). Although this appearance had reportedly been linked to non-neoplastic inflammation, we considered it potentially related to the subepithelial spread of the carcinoma due to its proximity to the main lesion.

Based on the preoperative examinations, including endoscopy, biopsy, and contrast-enhanced CT, the clinical diagnosis was as follows: M, post, 0-I, 25 × 25 mm, moderately to poorly differentiated adenocarcinoma, cT1, cN0, cM0, and cStage I [3]. The patient underwent laparoscopic distal gastrectomy (D1 + lymph node dissection) following the sixth edition of the Gastric Cancer Treatment Guidelines [4].

**Figure 2:**
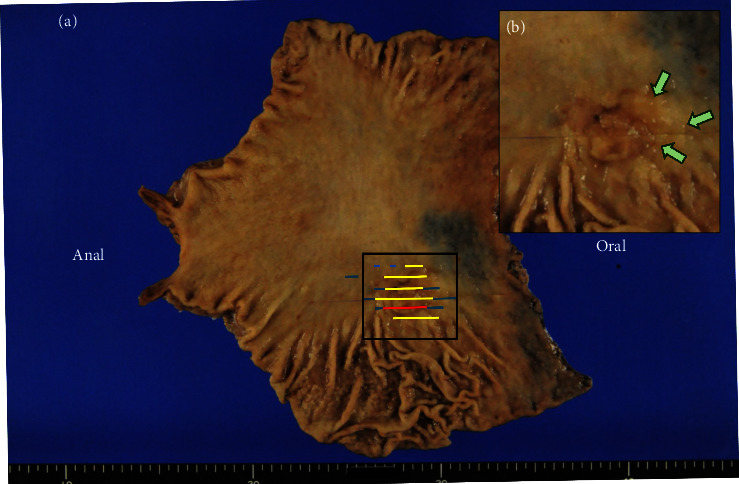
Resected specimen images: (a) a resected specimen showing a 47 × 32 mm protrusion on the posterior wall of the gastric body (both red and yellow lines indicate the protrusion). The microscopic image of the slice marked by the red line is shown in Figure 3. Blue lines indicate the area of subepithelial extension; (b) enlarged image of the black frame area in 2(a). White dots were seen on the oral side of the tumor.

**Figure 3:**
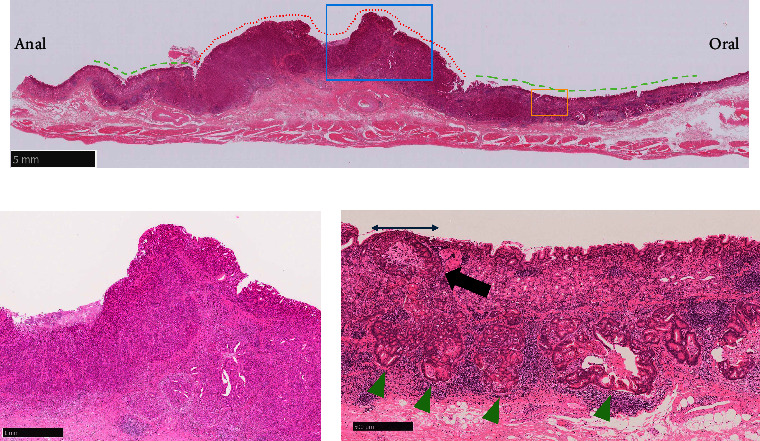
Histopathological images: (a) loupe image for the area indicated by the red line in Figure 2 (a). The carcinoma was exposed on the surface in the elevated area (red dotted line). By contrast, in the flat area (green dotted lines), the superficial layer was covered by non-neoplastic epithelium, and the carcinoma laterally extended within the submucosal layer (hematoxylin and eosin staining); (b) magnifying image within the blue box shown in 3(a). Nodules composed of irregularly shaped glands were observed in the deep area of the tumor (hematoxylin and eosin, ×20); (c) magnified image of the orange box in 3(a). In the flat area, relatively large irregularly shaped glands and cribriform structures were observed in the submucosal layer (green arrowheads). 500 *μ*m sized tumor nest was observed just below the epithelium (black arrow), at a distance of 50 *μ*m from the surface layer (hematoxylin and eosin, ×40).

The main lesion in the resected specimen was 47 × 32 mm, type 0-I (Figure 2(a), yellow and red lines). Microscopic evaluation revealed moderately differentiated tubular adenocarcinoma forming irregularly fused glands, invading the deep submucosal layer (SM2) (Figure 3(a)). The carcinoma formed a cribriform growth pattern in the deeper layer, making multiple nodules (Figure 3(b)).

**Figure 4:**
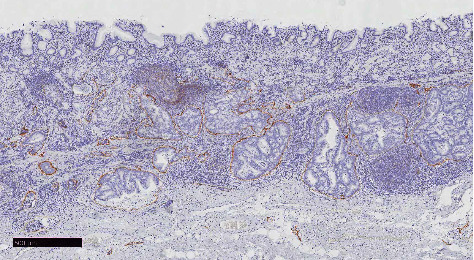
Pathological images with D2-40 staining, the image within the orange box shown in 3(a). The carcinoma nests were surrounded by the D2-40–positive cells, indicating lymphatic invasion in the area of lateral extent (D2-40, ×40).

**Figure 5:**
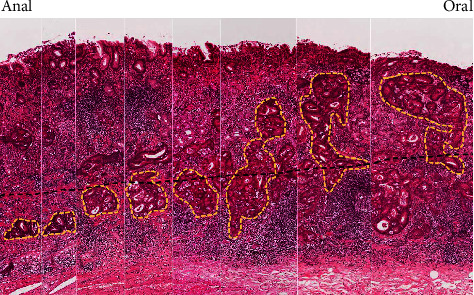
Sequential evaluation for a lymphatic vessel with carcinoma invasion. Sequential sections from the specimen delineated by the red line in Figure 2 confirmed the continuous extension of a lymphatic vessel with carcinoma (yellow dotted lines) from the submucosa to the mucosal lamina propria (left to right), reaching just below the surface. The black dotted line is an imaginary line for the muscularis mucosa (hematoxylin and eosin, ×100).

The white dots were macroscopically observed at the oral side of the elevated lesion (Figure 2(b), green arrows). In this flat area without superficial irregularity, carcinoma laterally extending along the submucosal layer was microscopically observed (blue lines in Figure 2, green dotted lines in Figure 3(a), and green arrowheads in Figure 3(c)). In the same area, tumor nests were surrounded by D2-40–positive cells, indicating lymphatic invasion (Figure 4). Numerous lymphoid follicles were observed around the carcinoma. Further sequential sections obtained from the same slice showed retrograde penetration of the carcinoma from the submucosa to the mucosal lamina propria along lymphatic vessels, with some vessels reaching just beneath the epithelium (black arrow in Figures 3(c) and 5). Despite comprehensive evaluations using Elastica Masson and desmin stainings, no disruptions of muscularis mucosa or submucosa were observed. Each tumor nested within a lymphatic vessel located near the epithelial surface was considered to appear as a WGA in endoscopic observation. There was no intraglandular necrotic debris (IND) or similar pathological findings in all sections.

The final histopathological diagnosis was moderately differentiated adenocarcinoma (M, post, Type 0-I, 47 × 32 mm, pT1b (SM 3500 *μ*m), INFb, Ly1c, V0, pN1, pPM0, pDM0, and pStage IB).

After surgery, the patient underwent regular follow-ups via endoscopy and CT scans. As of the latest follow-up, the patient has been alive for 2 years without recurrence.

## 3. Discussion

WGAs were first documented by Doyama et al. in 2015 and defined as “a white spherical appearance present just below the epithelium as seen by M-NBI.” WGAs indicate subepithelial necrotic tissues known as IND. The accuracy of gastric cancer using the WGA was reported to be 69.1%, demonstrating its complementary effect on the diagnosis [5].

Several changes can present as white and represent WGA-like findings related to proton pump inhibitor or vonoprazan usage, benign ulcers, low-grade adenomas, autoimmune gastritis, gastric lanthanum deposition, and gastric mucosa–associated lymphoid tissue lymphoma [6–10]. In contrast, debris accumulation within glandular lumens, as seen for the original endoscopic WGAs, is not uniformly white. In addition, mucus and epithelial cell deposits on noncancerous mucosa reported by Iwamuro et al. are not white despite showing WGA-like findings in endoscopy [6]. The mechanisms by which a material not inherently white appears white in endoscopy remain unknown.

Shimada et al. proposed a plausible hypothesis [11]. They reported a case of colonic pseudolipoma with pathologically aggregated small vacuoles in the mucosal layer that appeared white in endoscopy. They referred to the “white opaque substance,” which consisted of fat droplets on the mucosal surface layer, previously reported to appear white although they are inherently not white [12]. According to Shimada et al.'s hypothesis, light diffuse reflection occurs when substances with different refractive indices clump together in the shallow layers of the mucosa, resulting in the appearance of white.

Along with this hypothesis, the white color of WGAs can be derived from the diffuse reflection of light caused by the difference in the refractive indices of substances. In our case, intralymphatic invasion appeared white during endoscopy. In addition, considering the endoscopically visible depth of 80–100 *μ*m from the mucosal surface [11], vertically running lymphatic ducts filled with cancerous cells should appear as small round structures in endoscopy, which captures such ducts only within the limited range near the surface.

Although WGAs are usually observed in the marginal area within gastric cancers, WGA-like findings were found outside the cancer demarcation line in the present case. A comprehensive search on PubMed from 2015 to 2023 using the keywords “WGA” yielded no reports on WGAs or WGA-like findings stemming from the lymphatic invasion of cancer. Likewise, there have been no reports of lymphatic invasion with retrograde penetration from the submucosal layer to the mucosal lamina propria. However, lateral extension within the submucosa along lymphatic vessels is relatively common, suggesting that this phenomenon should occasionally occur with some frequency.

It is unclear why and how the lymphatic invasion in the submucosal layer developed retrograde penetration into the mucosal lamina propria in the present case. We hypothesized that the muscularis mucosas had occasional disruptions or weakening in the vicinity of the cancer, potentially facilitating communication between these layers. However, despite thorough pathological evaluations, no evident disruptions were observed. Although it was plausible that there was a previous ulceration, now healed or concealed by the cancer, we could not confirm this by using pathological evaluations. More cases are needed to elucidate the mechanism of this phenomenon.

In conclusion, we reported a case of gastric cancer with WGA-like findings indicating the intralymphatic extent of cancer beneath the epithelium. These findings can help in diagnosing the lateral extent of gastric cancers.

## Data Availability

The data that support the findings of this study are available from the corresponding author on request.
